# Comparative profiling of the transcriptional response to infection in two species of Drosophila by short-read cDNA sequencing

**DOI:** 10.1186/1471-2164-10-259

**Published:** 2009-06-07

**Authors:** Timothy B Sackton, Andrew G Clark

**Affiliations:** 1 Department of Molecular Biology and Genetics, Cornell University, Ithaca, NY 14853, USA; 2 Department of Organismic and Evolutionary Biology, Harvard University, 16 Divinity Ave, Cambridge, MA 02138, USA

## Abstract

**Background:**

Homology-based comparisons of the genes involved in innate immunity across many insect taxa with fully sequenced genomes has revealed a striking pattern of gene gain and loss, particularly among genes that encode proteins involved in clearing pathogens (effectors). However, limited functional annotation in non-model systems has hindered understanding of evolutionary novelties in the insect innate immune system.

**Results:**

We use short read sequencing technology (Illumina/Solexa) to compare the transcriptional response to infection between the well studied model system *Drosophila melanogaster *and the distantly related drosophilid *D. virilis*. We first demonstrate that Illumina/Solexa sequencing of cDNA from infected and uninfected *D. melanogaster *recapitulates previously published microarray studies of the transcriptional response to infection in this species, validating our approach. We then show that patterns of transcription of homologous genes differ considerably between *D. melanogaster *and *D. virilis*, and identify potential candidates for novel components of the *D. virilis *immune system based on transcriptional data. Finally, we use a proteomic approach to characterize the protein constituents of the *D. virilis *hemolymph and validate our transcriptional data.

**Conclusion:**

These results suggest that the acquisition of novel components of the immune system, and particularly novel effector proteins, may be a common evolutionary phenomenon.

## Background

Host-pathogen interactions are ubiquitous in nature, leading to coevolutionary dynamics that are predicted to drive rapid evolution of the immune system. It is now increasingly clear that this coevolutionary "arms race" leads to increased rates of protein evolution in genes encoding components of the immune system across a large number of taxa [1-7]. Recent work in mosquitoes [8,9] and fruit flies [5] has suggested that the immune system may also be unusual in the rate at which new genes are recruited into the system, and existing components of the system turn over by gene duplication or loss. Genes encoding effector proteins (proteins involved in bacterial killing and clearance), and particularly antimicrobial peptides (AMPs), often have lineage-restricted patterns of homology and show very rapid rates of gene turnover within gene families [5]. Most strikingly, two multigene families – the Drosomycin antimicrobial peptide family [10] and the Turandot family [11,12] of induced but otherwise uncharacterized proteins – appear to be evolutionary novelties restricted to *D. melanogaster *and related species in the *Sophophora *subgenus of drosophilids [5]. This pattern is in contrast to genes encoding components of immune-related signaling pathways, which are typically found as single copy orthologs even among distantly related insects [8,9,13], and have identifiable homologs in mammals [14]. Together, these observations suggest that disruption of stoichiometry, dosage, and other conserved interactions among signaling pathways is usually deleterious, leading to very low tolerance of gene copy variation among signaling pathways and preservation of single-copy orthologs across deep evolutionary time. Conversely, these observations suggest that pathway outputs retain flexibility, allowing novel effectors to be easily recruited into the system, potentially leading to rapid, and perhaps advantageous, proliferation of effector components across evolutionary time.

If this model is correct, effector proteins should be recruited and lost from the immune system at a relatively high frequency, which implies that novel components of the immune system remain to be discovered in species of *Drosophila *distantly related to *D. melanogaster*. It has long been recognized that highly divergent insect clades often harbour unique antimicrobial peptides: gambicin in mosquitoes [15], lebocin in *Bombyx *[16], thanatin from the bug *Podisus maculiventris *[17], and many others [reviewed in [18]]. However, the evolutionary dynamics of the acquisition of novel effector components in the innate immune system have not been considered previously. While the genome sequences of twelve *Drosophila *species [19] have allowed the discovery of many novel genes across this phylogeny, functional annotation still depends largely on homology to known *D. melanogaster *genes. Thus, our knowledge of the gain and loss of immune function genes has a strong ascertainment bias, and is restricted to genes that are paralogous to known genes in *D. melanogaster*.

In this study, we used short-read sequencing technology to characterize the transcriptional response to infection in *D. virilis*, a member of the *Drosophila *subgenus that last shared a common ancestor with *D. melanogaster *and the rest of the *Sophophora *subgenus 40 million years ago [20]. Short-read sequencing technology allows identification of differentially expressed genes without the need for prior annotations, and thus is ideal for detecting induced components of the *D. virilis *immune system that lack homologs in *D. melanogaster*. Here, we demonstrate that short-read sequencing of oligo(dT)-primed double-stranded cDNA provides a robust and accurate method to identify differentially transcribed regions of the genome. We then use this approach to sequence cDNA from infected and uninfected samples of *D. virilis *to characterize the genes that are induced by infection, and use that sequencing data to annotate novel components of the *D. virilis *immune system. Furthermore, using a LC-MS/MS proteomic approach, we identify immune-regulated protein constituents of the *D. virilis *hemolymph that validate novel transcripts that had been identified by the short-read cDNA sequencing as being responsive to infection in *D. virilis*.

## Results and discussion

### Aligning sequencing reads to the reference genome and identified genes regulated by infection

**Figure 1:**
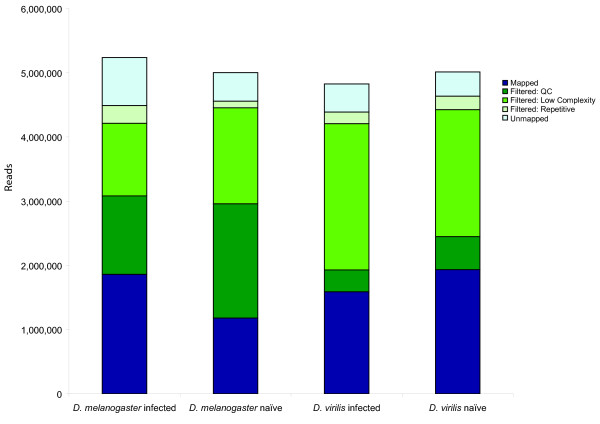
**Mapped and filtered sequencing reads**. Each bar shows the total number of sequencing reads obtained for each sample. The proportions successfully mapped to the reference genome are shown in dark blue, the proportions filtered are shown in shades of green (see legend), and the proportions that failed to map uniquely to the respective reference genome, but successfully passed all the filters, are shown in light blue.

We generated between 4.8 and 5.2 million 36 bp reads from one lane of Illumina/Solexa sequencing for each of four biological samples: naïve (uninfected) *D. melanogaster *iso-1 (*Dmel*U), 12 hours post-challenge (infected) *D. melanogaster *iso-1 (*Dmel*I), naïve (uninfected) *D. virilis *15010-1051.87 (*Dvir*U), and 12 hours-post challenge (infected) *D. virilis *15010-1051.87 (*Dvir*I). Infected flies were challenged by artificial infection with a mixed culture of one Gram-negative bacteria (*Serratia marcescens) *and one Gram-positive bacteria (*Enterococcos faecalis*); see Methods for details. Prior to mapping these sequencing reads to the reference genome, we filtered low complexity reads, low quality reads, and repetitive reads (including polyadenylated mRNA tails), resulting in between 1.6 and 2.6 million useable reads from each of the four samples (Figure 1; see Methods for details). To map reads to the reference, we used a combination of Mosaik (a BLAT-like tool optimized for aligning short-read sequencing reads to a reference; ) and BLAST [21], which allowed us to map between 71.3% and 83.7% of the reads that passed our filters, representing 1.2 to 1.9 million reads (Figure 1; see Methods for details). We then identified expressed regions in each species as described in the Methods. Overall, we identified 4,615 expressed regions in *D. melanogaster*; 3,001 of those regions were associated with a total of 2,540 annotated genes (Additional file 1). In *D. virilis*, we identified 6,737 expressed regions, of which 584 were associated with 579 genes (Additional file 2). Further details of our methodology are provided in the Methods.

Using a Hidden Markov Model (HMM), we assigned each expressed region to one of five states: strongly induced by infection, induced by infection, not regulated by infection, repressed by infection, or strongly repressed by infection. Each state is defined in the HMM by the binomial probability of observing the number of reads aligned to each base from the infected sample, given the total number of reads that align to each base; because the number of reads that map from the infected and naïve samples is not equal, the binomial probably for the unregulated class is not expected to be 0.50 (Table 1; see Methods for details). We infer that 841 (21.3% of all identified expressed regions) and 854 (13.2%) expressed regions are induced by infection in *D. melanogaster *and *D. virilis*, respectively (Table 2).

**Table 1:** Induced and strongly induced HMM classes

	*D. melanogaster*		*D. virilis*	
	Binomial probability	Median induction	Binomial probability	Median induction
Strongly induced	0.9034	5.60	0.9950	107.08
Induced	0.7078	1.68	0.7002	2.44
Not regulated	0.5722	1.10	0.4257	0.99
Repressed	0.4607	0.79	0.3170	0.64
Strongly repressed	0.1575	0.17	0.1766	0.35

Binomial probability of observing *x *infected reads aligned to a base, out of *n *total reads aligning to that base, estimated based on the HMM described in the text, and median corrected induction (log2 scale) for each class (see Methods for details).

**Table 2:** Number of expressed regions assigned to each induction class

	*D. melanogaster*	*D. virilis*
Strongly induced	107	33
Induced	747	808
Not regulated	1793	2684
Repressed	1280	2444
Strongly repressed	66	416
Not determined	622	352

### Validation of cDNA sequencing by comparison to published microarray data

**Figure 2:**
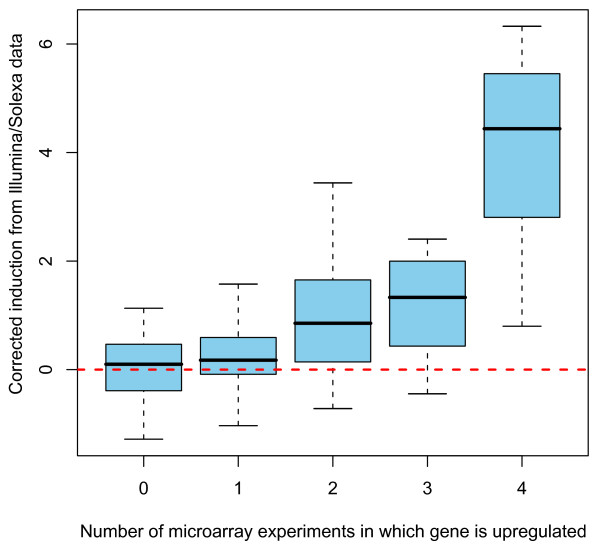
**Induction measured by short-read sequencing correlates with consistency of up-regulation in microarray data**. Boxplot of corrected induction (on a log2 scale) measured by short-read sequencing for genes detected as up-regulated in 0, 1, 2, 3, or 4 previous microarray studies that used a similar infection design (see text for details).

**Figure 3:**
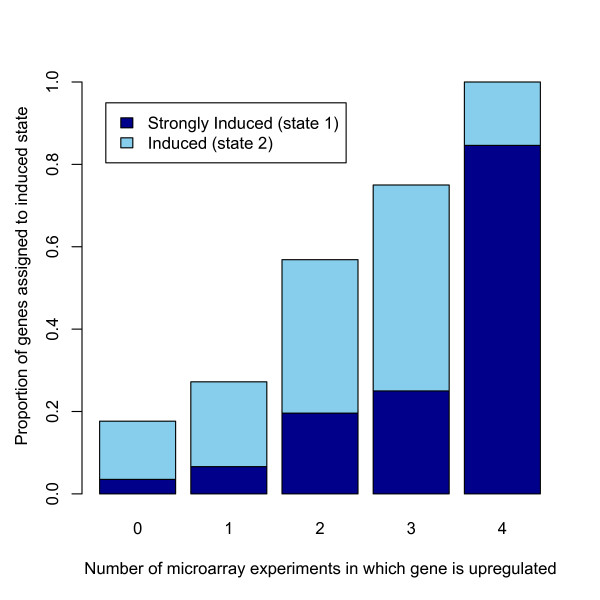
**Microarrays and short-read sequencing identify the same set of highly induced genes**. Each bar shows the fraction of genes detected as significantly up-regulated in either 0, 1, 2, 3, or 4 previous microarrays studies that are assigned to an induced state in our HMM model. The proportions that are assigned to the strongly induced state is shown in dark blue; the proportions that are assigned to the induced state is shown in light blue.

Because the transcriptional response to infection has been extremely well characterized in *D. melanogaster *[e.g. [22-26]], we can validate our short-read cDNA sequencing approach by comparison to previous studies. We compiled data from four microarray experiments published between 2001 and 2005 [22-24,26] that compared gene expression in infected and naïve *D. melanogaster *and characterized genes as up-regulated or down-regulated. Based on the definitions from each study, we count how many times a gene was defined as 'up-regulated' or 'down-regulated' across the four studies. There are 294 genes that are both present in our list of expressed regions and significantly regulated by infection on at least one of the four microarrays; those that are up-regulated in multiple microarrays are substantially more likely to be strongly induced in our data (Figure 2). Furthermore, genes induced in more microarrays are more likely to be assigned to an induced state by our HMM (Figure 3).

Additionally, we can quantitatively compare induction between our study and the previously published microarray where the infection protocol and sampling time post-infection where most similar to our study ([24]; in which *D. melanogaster *was infected by septic injury with a mixed bacterial culture and assayed at 12 hours post infection). Despite differences in the line (Oregon R vs. iso-1) and sex (male vs. female) of the flies, and the species and pathogenicity of bacteria used (non-pathogenic *E. coli *and *M. luteus *mixture vs. pathogenic *S. marcescens *and *E. faecalis *mixture), we still find a significant correlation between induction measured by microarray in DeGregorio *et al*. [24] and induction measured by our method (*r *= 0.3225, *P *< 2.2 × 10^-16^). However, this correlation is much weaker when limited to genes that are weakly induced (2-fold or less induction in both our data and the microarray data; *r *= 0.0429, *P *= 0.0388), and much stronger when limited to genes that are strongly induced (greater than 2-fold induction in both datasets; *r *= 0.5053, *P *= 0.002). While these results demonstrate considerable consistency between induction measured by short-read sequencing of oligo(dT) primed, double-stranded cDNA and induction measured by traditional microarray methods, they also suggest that higher depth of coverage may be needed to accurately assay weakly induced genes. However, at least for identifying strongly induced genes, short read sequencing approaches appear to be robust and accurate, suggesting that this approach may prove to be a simple and cost-effective way to identify differentially regulated genes in poorly annotated genomes in response to any number of treatments of biological interest.

### The transcriptional response to infection in *D. virilis *and *D. melanogaster*

We identified 841 expressed regions that appear to be induced by infection in *D. virilis*. Because of the 3' bias in our cDNA preparation, the relatively low coverage of our sequencing, and the lack of annotation of 3' UTR sequence in the *D. virilis *genome, only about 5% of these induced regions overlap with an annotated exon. To attempt to associate a greater percentage of induced regions with genes, we analyzed the genomic region in more detail for these 841 regions, and preliminarily assigned expressed regions to annotated gene models if they were fewer than 500 bp from the 3' end of the nearest gene model, and more than 1 kb from the 3' end of all other gene models (see Methods for details). We eliminated from further analysis induced regions (but not strongly induced regions) located on minor scaffolds (< 1 Mbp), leaving a total of 199 candidate induced regions in *D. virilis*, 101 of which can be preliminarily associated with 95 *D. virilis *genes.

In order to understand the similarities between the *D. melanogaster *and *D. virilis *immune responses, we focused on the 490 genes in *D. melanogaster *(Additional file 3) and the 95 genes in *D. virilis *(Additional file 4) associated with induced regions. We used three approaches to identify orthologs and paralogs of these gene models. First, for any gene model included in the manual homology annotation of immune system genes [5], we used the homology and orthology assignments from that work. For the remaining genes, we used homologs annotated by FlyBase. If FlyBase reported no homolog, we verified the absence of homologs by reference to the homology assignments generated by the *Drosophila *12 Genomes Consortium [19].

Of the 490 induced genes in *D. melanogaster *(Additional file 3), 19 have no identifiable homologs in *D. virilis*, 444 have homologs in *D. virilis*, and 27 have ambiguous homology. Genes associated with expressed regions assigned to state 1 (highly induced) are significantly more likely to lack homologs in *D. virilis *that genes associated with expressed regions assigned to state 2 (Odds ratio = 5.22, Fisher's Exact Test *P*-value = 0. 001). As highly induced genes are much more likely to represent effectors, this result is expected based on the analysis of Sackton *et al*. [5], which showed that effector proteins are much more likely to have lineage restricted patterns of homology than immune system genes as a whole.

This same pattern seems to hold in *D. virilis*, suggesting that the high rate of turnover in effector proteins may be quite general. In *D. virilis*, the 95 gene models associated with induced regions (Additional file 4) include 8 with no identifiable homologs in *D. melanogaster *and 87 with homologs in *D. melanogaster*. Like in *D. melanogaster*, genes associated with expressed regions assigned to state 1 are more likely to lack homologs that those associated with expressed regions assigned to state 2, although this pattern is not significant (Odds ratio = 3.08, Fisher's Exact Test *P*-value = 0.15).

In both species, gene models that are highly induced (state 1) encode significantly shorter predicted polypeptides than induced (state 2) gene models (Mann-Whitney U; *D. melanogaster *P = 1.7 × 10^-9^, *D. virilis *P = 2.3 × 10^-4^). Furthermore, in both species gene models that lack homologs in the other species are significantly shorter than those gene models that have homologs (Mann-Whitney U; *D. melanogaster *P = 7.4 × 10^-7^, *D. virilis *P = 0.006). Because it can be difficult to infer homology patterns between highly divergent short peptides, we cannot rule out the possibility that the deficiency of gene models with detectable homologs among the highly induced class reflects high rates of divergence rather than lack of homologs, although the observation that immune effector proteins tend to be highly conserved at the protein sequence level among Drosophilids [5] suggests against this interpretation.

The putatively induced genes that have identifiable homologs between species reveal broad similarities in the transcriptional response to infection between *D. melanogaster *and *D. virilis*. As expected based on our comparison of the *D. melanogaster *induced genes to previous microarray studies, most of the highly induced genes are antimicrobial peptides, Turandots, and other immune-induced peptides such as the IMs [Drosophila immune molecule, [27]]. We also see other immune genes such as *PGRP-SB1*, *Transferrin 1*, and *TepII *strongly induced after infection in *D. melanogaster*. In *D. virilis*, homologs of many effectors or putative effectors are identified as induced in our sample. The genes associated with expressed regions assigned to state 1 in *D. virilis *encode Attacins, Cecropins, Metchnikowin, Diptericins, PGRP-SB1, and a protein with homology to IM1. We also find evidence for induction of homologs of two additional peptidoglycan recognition proteins (*PGRP-LB *and *PGRP-LC*) and the *D. virilis *homologs of *Relish*, *MP1*, and *necrotic *(Additional file 4). Given the limitations of our sequencing strategy in detecting weakly induced genes, we have almost certainly not detected all induced signalling and recognition genes in *D. virilis*. Thus, in the next section, we focus on the most strongly induced category of genes in both species, the AMPs.

### Differences in the members of AMP families induced after infection

Despite the overall similarity of the transcriptional response to infection in *D. virilis *and *D. melanogaster*, notable differences exist in the pattern of induction of members of AMP gene families. The *D. melanogaster *genome encodes 20 antimicrobial peptides that are members of seven gene families. These peptides can be broadly grouped into three categories: cysteine-rich peptides characterized by pairs of disulfide bonds (Defensin: Def; and Drosomycins: Drs, Drs-l, Dro2, Dro3, Dro4, Dro5, Dro6), peptides with an amphiphilic α-helical conformation (Cecropins: CecA1, CecA2, CecB, CecC), and proline or glycine rich peptides (Attacins: AttA, AttB, AttC, AttD; Drosocin: Dro; Metchnikowin: Mtk, and Diptericins: Dpt and DptB). Five of these families – Diptericins, Cecropins, Attacins, Metchnikowin, and Defensin – have homologs in *D. virilis*, encoding total of 15 known antimicrobial peptides; Drosocin and Drosomycins are absent from the entire *Drosophila *subgenus [5].

**Figure 4:**
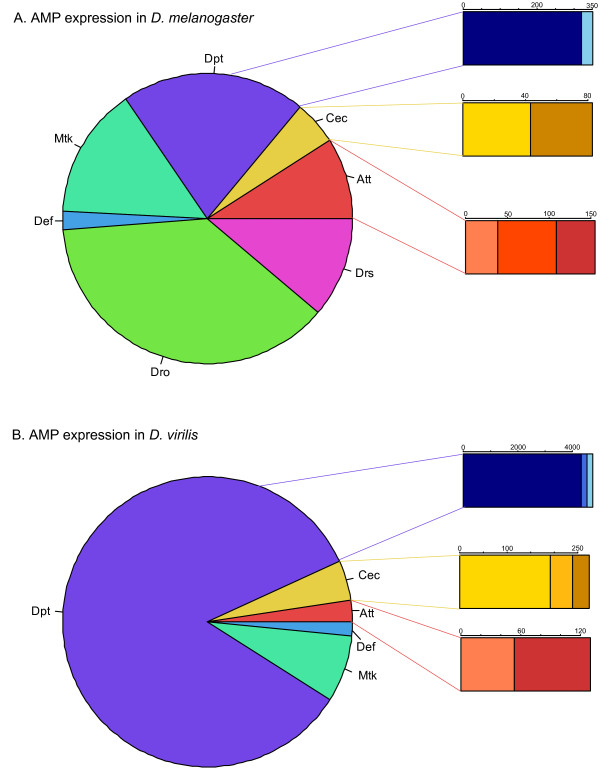
**Relative expression of antimicrobial peptides in *D. melanogaster *and *D. virilis *after infection**. Each pie chart shows the fraction of total reads from the infected samples that map to AMPs that are represented by a given peptide family. Each bar chart shows the reads mapped to individual families from the infected samples, with different colours representing individual members of each AMP family. A) *D. melanogaster*; B) *D. virilis*.

Most of these AMPs are strongly induced after infection (Table 3 and Table 4). In *D. virilis*, 10 of the 15 AMPs are highly induced (state 1) after infection; the remaining 5 are not expressed in our sample. In *D. melanogaster*, 10 of the 20 AMPs are highly induced, an 11^th ^is moderately induced (state 2), and a 12^th ^appears to be very weakly expressed, but not induced (state 3). Among the homologous AMP families, the Diptericins are the most strongly induced in both species: marginally so in *D. melanogaster *(Table 3), and strikingly so in *D. virilis *(Table 4). Furthermore, in both species Diptericins represent the largest fraction of total AMP transcription in the infected sample (as measured by average coverage of the infected sample), and Diptericin expression is dominated by a single paralog (Figure 4), although when non-homologous AMP families are included, Dro dominates overall transcription in the infected *D. melanogaster *sample. The extent to which one AMP predominates is strikingly different between species, however: in *D. melanogaster*, Dro, Mtk, Drs and Dpt are all transcribed at high levels in the infected sample, whereas no other AMP is transcribed at nearly the level of Dpt in *D. virilis *(Figure 4), suggesting that the spectrum of antimicrobial peptides produced after infection may be narrower in *D. virilis *than in *D. melanogaster*. There are also differences in the relative transcription level of paralogs within the Cecropin, Attacin, and Diptericin AMP families. In the *D. virilis *sample, one member of each family tends to dominate transcription (Figure 4 and Table 4), whereas in *D. melanogaster *the relative transcription of paralogs within a AMP family is less skewed (with the exception of Dpt; Figure 4 and Table 3).

**Table 3:** Induction of antimicrobial peptides in *D. melanogaster*.

AMP family	*D. melanogaster *gene	HMM state	Induction	Average Coverage (Naïve)	Average Coverage (Infected)
Attacins	AttA	strongly induced	21.59	1.33	38.37
	AttB	strongly induced	23.23	2.26	70.20
	AttC	strongly induced	35.90	0.96	46.29
	AttD	ND^1^			
Cecropins	CecA1	strongly induced	27.33	1.18	43.20
	CecA2	strongly induced	43.80	0.68	39.59
	CecB	ND			
	CecC	ND			
Defensins	Def	moderately induced	3.35	7.68	34.40
Diptericins	Dpt	strongly induced	80.26	2.99	320.51
	DptB	strongly induced	Inf	0.00	30.37
Metchnikowin	Mtk	strongly induced	23.47	7.94	249.23
Drosocin	Dro	strongly induced	17.07	28.03	640.13
Drosomycins	Drs	strongly induced	6.99	19.28	180.39
	Dro2	ND			
	Dro3	ND			
	Dro4	not regulated	0.95	12.74	10.00
	Dro5	ND			
	Dro6	ND			
	Drs-l	ND			

^1^Not Detected in our sample.

**Table 4:** Induction of antimicrobial peptides in *D. virilis*.

AMP family	*D. virilis *gene	*D. melanogaster *homolog	HMM state	Induction	Average Coverage (Naïve)	Average Coverage (Infected)
Attacins	dvir_GLEANR_6000	AttA/AttB	strongly induced	Inf	0.00	53.43
	dvir_GLEANR_6001	AttA/AttB	strongly induced	329.41	0.31	76.45
	dvir_GLEANR_6553	AttC	ND^1^			
	dvir_GLEANR_8042	AttD	ND			
Cecropins	dvir_GLEANR_10332	CecA1/CecA2/CecB/CecC	strongly induced	94.93	2.73	191.95
	dvir_GLEANR_10659	CecA1/CecA2/CecB/CecC	strongly induced	52.34	1.20	46.65
	dvir_GLEANR_10661	CecA1/CecA2/CecB/CecC	strongly induced	Inf	0.00	35.45
	dvir_GLEANR_10660	CecA1/CecA2/CecB/CecC	ND			
	dvir_GLEANR_10662	CecA1/CecA2/CecB/CecC	ND			
Defensins	dvir_GLEANR_7763	Def	strongly induced	147.31	0.78	85.64
	dvir_GLEANR_6510	Def	ND			
Diptericins	dvir_GLEANR_5386	Dpt	strongly induced	326.80	0.88	212.92
	dvir_GLEANR_5385	Dpt	strongly induced	3218.05	1.81	4322.99
	dvir_GLEANR_5387	DptB	strongly induced	317.73	0.89	209.23
Metchnikowin	dvir_GLEANR_7753	Mtk	strongly induced	58.15	9.29	400.44

^1^Not Detected in our sample.

On a broader scale, in both species the proline- and glycine- rich peptides represent most of the total AMP transcription (*D. virilis*: 93.6%, *D. melanogaster*: 81.9% of the total coverage across all AMPs, normalized for length). Again, though, *D. melanogaster *appears to transcribe a broader spectrum of antimicrobial peptides in response to infection, with a substantial fraction of the total transcription of AMPs in *D. melanogaster *associated with cysteine-rich (Drs/Def; 13.5%) AMPs. This analysis of course excludes any uncharacterized AMPs in *D. virilis*. However, as discussed below, the most promising candidates for novel *D. virilis *AMPs appear to be in the glycine- and proline- rich family, suggesting that *D. virilis *may in fact produce a narrower range of AMP types in response to our artificial infection protocol. In is possible that this observation could be attributable to differences in the ability of the bacterial species used to replicate efficiently between *D. virilis *and *D. melanogaster*, resulting in differences in the nature of the infection experienced by the two species of *Drosophila*. Alternatively, D. *virilis *and *D. melanogaster *differ at a number of ecological traits, any of which could potentially lead to different selective pressures for the diversity of AMPs produced: *D. melanogaster *is tropical, *D. virilis *is Holarctic; *D. melanogaster *breeds on a wide range of substrates, typically rotting fruit, *D. virilis *breeds on sap fluxes [20]. While it is tempting to speculate, a fuller understanding of the diversity of *D. virilis *AMPs and the persistence of differences in transcription in response to multiple challenges will be needed before the hypothesis that *D. virilis *responds to infection with a narrower and less diverse range of AMPs can be established.

### Novel components of the *D. virilis *immune system

As noted above, a number of *D. virilis *gene models associated with induced regions do not have identifiable homologs in *D. melanogaster *or other species of the *melanogaster *species group. In this section, we discuss these putative novel components of the *D. virilis *immune system in more detail (Table 5). Broadly speaking, these ten gene models fall into three classes: those encoding predicted proteins that are apparently not secreted (lack a signal peptide); those encoding predicted proteins that are short, secreted, negatively charged, and appear to be distantly related to the IM proteins in *D. melanogaster*; and those encoding short, positively charged, secreted proteins that are often proline or glycine rich and may represent novel AMPs. Predicted proteins were considered secreted if SignalP [28] predicted a signal peptide.

**Table 5:** Genes associated with induced regions in *D. virilis *that lack homologs in *D. melanogaster*.

*D. virilis *gene model	Induction State	Induction	Signal Peptide?	Size (kD)	Net Charge
GF_DGIL_SNO_29059273/GF_DGIL_SNO_29059274*	1	716.86	yes	4.13	+1
dvir_GLEANR_6300	2	1.55	yes	4.1	+1
dvir_GLEANR_7739	1	Inf	yes	6.13	+9
dvir_GLEANR_5464	1	61.95	yes	4.71	+4
dvir_GLEANR_3774	2	2.89	yes	4.57	+3
dvir_GLEANR_345	1	26.91	yes	2.23	-2
dvir_GLEANR_5361	2	3.14	yes	2.22	-1
dvir_GLEANR_15023	2	2.22	no		
dvir_GLEANR_13841	2	2.04	no		

*These gene models are paralogs

For proteins with a predicted signal peptide, molecular weight and net charge of the predicted mature peptide were computed using the ExPASy web server.

#### Gene models lacking a signal peptide

Two putatively induced *D. virilis *gene models lack a signal peptide. One, dvir_GLEANR_13841 is a short protein (155 aa) that is moderately induced (corrected induction 2.04, assigned to state 2). The second, dvir_GLEANR_15023, is somewhat longer, 280 amino acids, but is highly repetitive, consisting of 20 repeats of a 12–17 amino acid motif. The repetitive nature of this predicted gene makes identifying putative homologs difficult; we fail to detect any via BLAST, and no homologs in any species are reported in *Drosophila *12 Genomes Consortium [19]. This gene model is flagged as potentially representing a repeat-contaminated gene model, suggesting that this result may be artefactual.

#### Secreted, IM-like peptides

Two gene models in *D. virilis *that are putatively associated with an induced region, dvir_GLEANR_345 and dvir_GLEANR_5361, have strong evidence for a signal peptide, and are short (fewer than 50 amino acids), suggesting the possibility that these are novel effector proteins. However, they are unlikely to be antimicrobial peptides. Almost all antimicrobial peptides have a net positive charge; the predicted proteins encoded by these two GLEANR models both have a negative net charge. The proteins with the closest homology are the IM proteins of *D. melanogaster*. These are a family of short, strongly induced peptides of unknown function.

#### Putative novel AMPs

The remaining three GLEANR models, plus the two non-GLEANR gene models (which are paralogs of each other) that appear to be strongly induced by infection, are all secreted peptides with predicted molecular weights between 4 and 6 kD and predicted positive charge at physiological pHs (Table 5). All three have homologs in *D. mojavensis *and *D. grimshawi*, the two species with sequenced genomes that are most closely related to *D. virilis*, but not in any other species of *Drosophila *with sequenced genomes. In addition dvir_GLEANR_3774 and the non-GLEANR gene model are both ~18% proline, which combined with their patterns of induction, size, and charge suggests that they might be similar in function to the proline-rich family of AMPs, including abaecins and apidaecins from bees (~30% proline), as well as Mtk and Dro from *Drosophila *(~25% proline).

### Protein constituents of the *D. virilis *hemolymph

In order to further characterize the *D. virilis *immune response, we used a proteomic approach (iTRAQ/tandem MS) to analyze the protein constituents of *D. virilis *hemolymph, in both naïve (uninfected) and immune-challenged flies. Previous work in *D. melanogaster *has focused on identifying proteins that increase in concentration in the hemolymph after immune challenge [27,29-32], characterizing the proteins involved in clotting [33,34], and mapping the proteins present in larval hemolymph in an unchallenged state [35,36]. This wealth of information about the protein constituents of hemolymph in *D. melanogaster *thus provides a comparative context for proteomic studies of other species of *Drosophila*.

In order to characterize the protein content of the *D. virilis *hemolymph, we used two methods. First, we assessed the relative concentration of each identified protein using the emPAI statistic implemented in MASCOT [37]. This statistic is calculated based on the fraction of potentially observable peptides that are in fact observed in the sample for any given protein. Second, we used the iTRAQ chemistry (Applied Biosystems), which uses tags to mark each sample (in this case, infected and uninfected hemolymph), to estimate the relative abundance of each identified protein in the infected and uninfected samples using the software ProteinPilot (Applied Biosystems).

**Figure 5:**
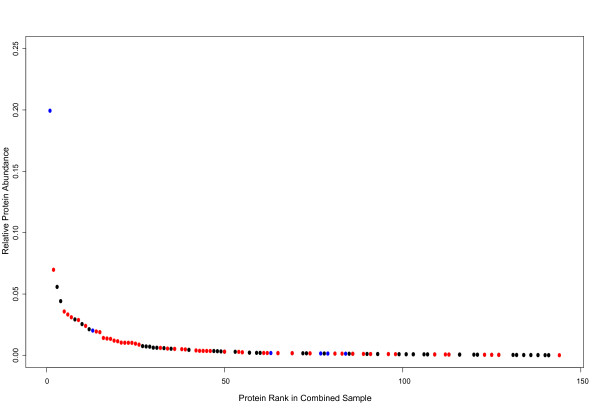
**Distribution of protein abundance in *D. virilis *hemolymph**. Plot of ranked protein abundance (see Methods for details) of proteins detected in the hemolymph of *D. virilis*. Points are coloured based on homology to *D. melanogaster *and function: red points represent proteins with *D. melanogaster *homologs that have been reported to be present in *D. melanogaster *hemolymph, black points represent proteins with *D. melanogaster *homologs that have not been reported to be present in *D. melanogaster *hemolymph, and blue points represent proteins without *D. melanogaster *homologs.

In general, the protein constituents of the *D. virilis *hemolymph show substantial homology to previously identified components of the *D. melanogaster *hemolymph (Figure 5). In the total (combined infected and naïve) sample, 47.3% of the total protein is composed of *D. virilis *proteins with *D. melanogaster *homologs that have been identified as present in the *D. melanogaster *hemolymph. Another 30.2% of the total protein in *D. virilis *hemolymph is consists of proteins with *D. melanogaster *homologs that have not been previously characterized as components of the *D. melanogaster *hemolymph. Finally, 22.6% of the *D. virilis *hemolymph is composed of proteins without *D. melanogaster *homologs. Surprisingly, one of these proteins (FBpp0227890) represented 20% of the total protein content of the *D. virilis *hemolymph; this protein has identifiable homologs in *D. mojavensis *and *D. grimshawi*, but no other species of insect with sequenced genomes, and has no significant BLAST similarity to any other proteins in Genbank. Thus, while the most common protein in *D. virilis *hemolymph appears to be an evolutionary novelty, the general spectrum of hemolymph proteins between *D. melanogaster *and *D. virilis *appear broadly conserved.

**Figure 6:**
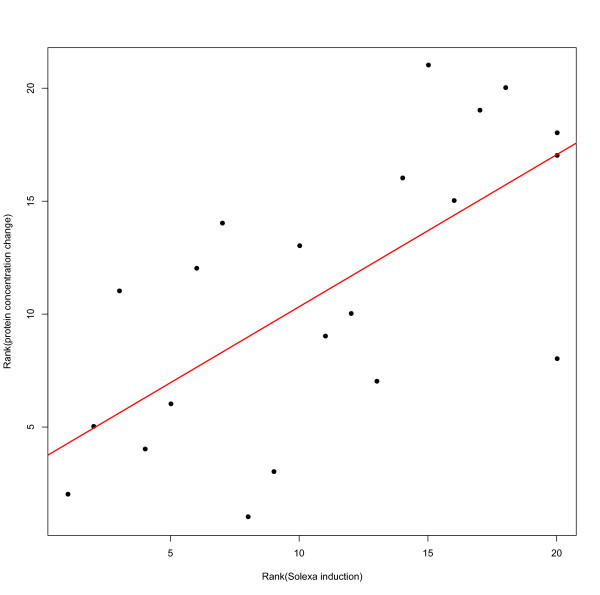
**Correlation between change in protein concentration in the *D. virilis *hemolymph after infection and transcriptional induction of the corresponding transcript after infection**. Each point represents a protein with data on both transcript and protein abundance. Ranked change in protein concentration after infection is plotted on the Y-axis, and ranked change in transcript abundance after infection is plotted on the X-axis. Spearman's ρ = 0.672, *P *= 0.0008.

The changes in protein concentration in the *D. virilis *hemolymph we observe after infection are largely consistent with our expectations based on our transcriptional data. We see a significant correlation between transcriptional induction and changes in protein concentration (Spearman's ρ = 0.672, *P *= 0.0008; Figure 6), although only a small fraction of the total expressed regions we observe in our transcriptional data can be associated with proteins identified in the *D. virilis *hemolymph. Consistent with our observation that genes encoding homologs of Diptericin are the most strongly induced AMPs in *D. virilis *(Figure 4B), we find that Diptericin homologs are also the proteins that increases most in concentration after infection (Additional file 5; note that the Dpt homologs FBpp0234332 and FBpp0234333 cannot be distinguished at the protein level in *D. virilis*). Finally, one of the putative novel AMPs identified in our transcriptional analysis (dvir_GLEANR_7739, encoding FBpp0236872) is the protein that increases in concentration second most in our hemolymph sample (after the Dpt homologs FBpp0234332/FBpp0234333), providing further evidence for a potential antimicrobial or immune role for this protein.

## Conclusion

In this study, we used short read cDNA sequencing to characterize the transcriptional response to infection in *D. virilis *and *D. melanogaster*. We show that even a relatively small number of sequencing reads (1 lane per sample, about 5 million reads before filtering and about 1.2 million mapped reads) can produce reliable estimates of induction, at least for strongly induced genes, consistent with recent results suggesting high technical repeatability of this approach [38]. By comparing the relative induction of AMP gene families in *D. melanogaster *and *D. virilis*, we show that significant differences in the relative induction of different peptides exist between species. The striking increase in induction of Dpt homologs in *D. virilis *relative to *D. melanogaster *is supported by the observation that Dpt homologs represent 11.5% of the protein in *D. virilis *hemolymph from infected flies, far higher than any other AMP. Finally, we show that some predicted *D. virilis *genes that lack homologs to *D. melanogaster *share characteristics with the proline-rich AMP superfamily, and the protein encoded by at least one of these genes is detectable in the hemolymph of infected flies, suggesting that *D. virilis *likely possesses lineage-restricted immune system components, and that the pattern we observe in *D. melanogaster *is general. Taken together, these results suggest that novel downstream components of the immune system can be rapidly integrated of relatively short time scales. The adaptive potential of gene gain and loss should not be overlooked in the evolutionary dynamics of host immune systems.

## Methods

### Biological samples for transcriptional analysis

The *Drosophila *stocks used in this experiment were the sequenced strains of *D. melanogaster *(iso-1) and *D. virilis *(15010-1051.87). Flies were maintained in bottle cultures on a rich dextrose medium at 25° and in 12 hr:12 hr light/dark for the duration of the experiment. We infected 50 females of each species with a mixed bacterial culture of *Serratia marcescens *and *Enterococcos faecalis *by pricking the thorax with a 0.1-mm dissecting pin (Fine Science Tools, Foster City, CA) dipped in bacterial culture, as previously described [39]. We chose to use a mixed culture, consisting of one Gram-negative bacteria and one Gram-positive bacteria, in order to maximize our ability to detect a diversity of immune-inducible proteins. At 12 hours after infection, infected flies and a sample of 50 naïve flies were frozen in liquid nitrogen. We extracted total RNA from frozen flies using standard protocols (Trizol). After extraction, total RNA was treated with DNase to remove potential genomic DNA contamination, according to the manufacturer's protocols. We synthesized first strand cDNA using oligio-d(T) primers, and then synthesized second strand cDNA, according to standard protocols. Sequencing was done by the Cornell University Life Sciences Core Laboratories Center on an Illumina GA2 sequencer.

### Aligning reads to the reference genome

Prior to mapping reads to the reference genome, we filtered low quality, low complexity, and repetitive reads. We first removed any read having fewer than 24 bases with a Phred quality score (Q) greater than 20; this is our 'low quality' filter. Next, we removed any read with low nucleotide complexity (80%+ of the sequence composed of only 2 bases) or repetitive elements (more than half the sequence composed of dinucleotide or trinucleotide repeats). Finally, we removed any reads with a mononucleotide run greater than 24 bases.

After filtering, we did an initial round of mapping to the repeat-masked *D. virilis *or *D. melanogaster *reference genome with Mosaik, a software program written by the Gabor Marth lab (A. Quinlan and G. Marth, , unpublished) that uses a BLAT-like approach. The program hashes the genome into unique *n*-mers (where *n*, the hash size, can be specified by the user; we used 17 bp), which it uses as seeds to align the sequencing reads to the reference. We required all matches to align for at least 91% of the read length (33 of 36 bp) and have no more than 3 mismatches.

To supplement the mapping from this initial Mosaik run, we took two approaches. First, we noticed that some reads fail to map because low quality ends or partial polyA sequence cause them to fail to pass our alignment length filter. In order to get around this, we trimmed up to 10 bp from the end of any read where average quality across a 5 or 10 bp segment was less than Phred Q20. We also trimmed any mononucleotide run from the end of a read. After trimming, we rejected any read that was shorter than 20 bp, or that failed to pass our QC filters (which we reran on the trimmed sequence). The remaining reads were then rerun in Mosaik, using the same parameters described above.

Finally, we attempted to map the remaining sequences using BLAST. Any reads that passed all our quality control filters, but could not be mapped using Mosaik even after end-trimming, were run through a BLAST pipeline: we used blastn with a word size of 7 and an E-value cutoff of 1 × 10^-6^, and considered any read mapped if either 1) there was only a single BLAST hit to the reference genome, or 2) there were fewer than 10 hits and the best hit aligned over at least 90% of the read length and had a lower E-value than the next best hit. Any read with more than 10 hits was considered repetitive and was not mapped.

After mapping, we combined the output from both Mosaik runs and the BLAST pipeline to produce a single file for each contig containing the depth of coverage at each base in the genome (using the program ace2dep, from the Marth lab, to convert Mosaik output into depth, and custom Perl scripts to convert BLAST output into depth information). The depth of coverage at each base along a scaffold was then the input to our pipeline to identify expressed regions of the genome regulated by infection.

### Identifying regions regulated by infection

**Figure 7:**
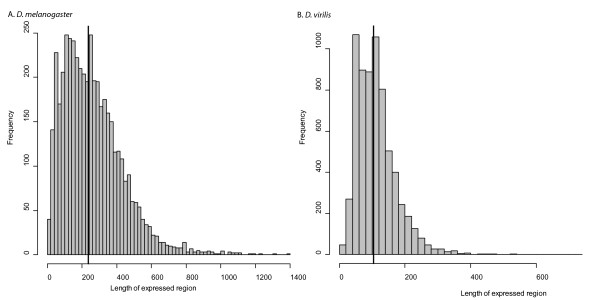
**Distribution of lengths of expressed regions**. Histogram of the lengths of expressed regions in A) *D. melanogaster *and B) *D. virilis*. Solid black lines show the median of each distribution.

We first defined an expressed region as any contiguous stretch of DNA along a scaffold where the minimum coverage of the combined infected and uninfected samples at any one base is 1, and the average combined coverage across the region is at least 10. Based on this definition, we identified 4615 expressed regions in *D. melanogaster *(Additional file 1) and 6737 in *D. virilis *(Additional file 2). The median length of an expressed region in *D. melanogaster *is 237 bp (Figure 7A), compared to a median length in *D. virilis *of 104 bp (Figure 7B). Approximately the same number of reads map to *D. virilis *and *D. melanogaster *(Figure 1), so it is unclear why we identify more regions that are on average shorter in *D. virilis*.

To determine the extent to which each expressed region responds to infection, we developed a Hidden Markov Model, with five hidden states representing the degree of induction (highly induced, moderately induced, unchanged, moderately repressed, highly repressed), where the emission probability for each state is the binomial probability of observing *x *infected coverage given *n *total coverage at each base pair, and the observed data is the coverage of infected reads at each base. We used the HiddenMarkov package in R to optimize our HMM using the Baum-Welch algorithm, and to calculate the most probable set of states using the Viterbi algorithm. The optimized emission probabilities for each state are given in Table 1. Before running the HMM, we removed sites with less than 10× coverage pooled across samples, as there is very little power to distinguish between states with so few reads. In order to increase the number of expressed regions for which all sites are assigned to the same state, we tuned the transition probabilities by increasing the probability of remaining in the same state, and decreasing the probabilities of transitioning between states proportionally, so that the highest probability in the matrix was equal to 0.999. Empirically, this tuning appeared to increase the consistency of our results, with fewer expressed regions being assigned to multiple states.

To determine the most likely state for any given expressed region, we weighted the Viterbi estimate of the state of each base in an expressed region by the summed coverage of that base. If one state had a majority of this measure, we assigned the expressed region to that state. For the 5.2% of expressed regions in *D. virilis *and 13.5% of expressed regions in *D. melanogaster *for which either the weighted sum assigned to the best state was not at least 50% of the total weighted sum, or for which no single base had sufficiently high coverage to be included in our HMM, we consider the state as "not determined" (Table 2).

### Associating expressed regions with genes

**Figure 8:**
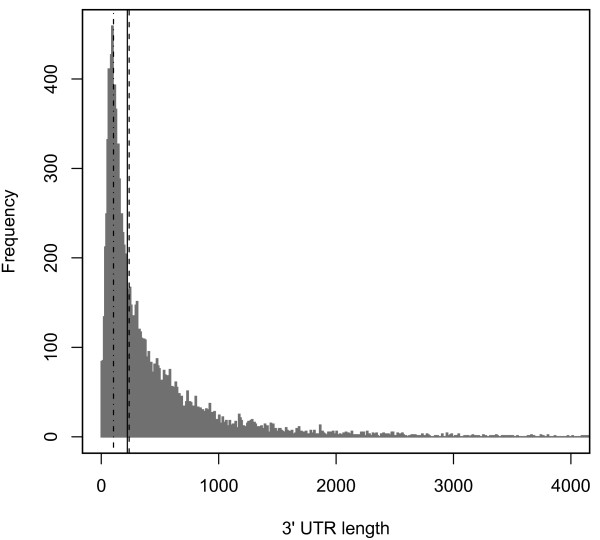
**Distribution of 3' UTR length in *D. melanogaster***. Histogram of the lengths of annotated 3' UTRs in *D. melanogaster*. The solid black line is the median of the distribution; the dashed line is the median length of expressed regions in *D. melanogaster*; and the dot-dashed line is the median length of expressed regions in *D. virilis*.

We used two methods to associate expressed regions with annotated genes. First, we used an automated first-pass method, where we simply asked whether any base in an expressed region also falls into an annotated exon, based on the *D. melanogaster *release 5.7 annotations and the *D. virilis *release 1.1 annotations available as GFF files from FlyBase (downloaded 4/24/2008). However, given the 3' bias inherent in oligo(dT) primed cDNAs, plus the relatively low coverage to which we sequenced, our identified expressed regions are short (Figure 8). In *D. melanogaster *this does not pose much of a challenge, as the genome annotation is quite mature and includes fully annotated 3' UTRs. In *D. virilis*, however, 3' UTRs are generally not annotated, leading to any expressed region that falls entirely in a UTR failing to be associated with any gene. Given the length distribution of 3' UTRs in *D. melanogaster *(Figure 8), we expect a substantial fraction of our expressed regions in *D. virilis *to suffer from this problem.

As a partial remedy, we analyzed the 841 induced genomic regions in *D. virilis *in more detail. For these 841 regions, we extracted the nearest 3' end of a gene to the start of the expressed region on the positive strand and the nearest 3' end of a gene to the end of the expressed region on the negative strand. We consider one of these expressed regions to be putatively associated with a annotated gene in *D. virilis *if the smaller distance was less than 500 bp and the larger distance was greater than 1 kb, or if the smaller distance was less than 200 bp and the larger distance was greater than 500 bp. Regions more distant that 500 bp from any other gene were declared "putatively unassociated," and the remaining regions were declared "ambiguous." The number of regions assigned to each class is listed in Table 6.

**Table 6:** Association of induced expressed regions with gene models in *D. virilis*.

	Strongly Induced (state 1)	Induced (state 2)
Overlap GLEANR model	10 (30.3%)	34 (4.21%)
Associate with GLEANR model	11 (33.3%)	52 (6.44%)
Ambiguous	1 (3.03%)	31 (3.84%)
Not associated with annotated GLEANR model	11 (33.3%)	691 (85.5%)

Total Regions	33	808

In order to understand why there is a large difference in the fraction of expressed regions that can be associated with annotated genes between the two induced classes in *D. virilis*, we divided the *D. virilis *scaffolds into "major" scaffolds (the 23 scaffolds with at least 1 Mb of sequence, which represent 77% of the total *D. virilis *sequence) and the remaining "minor" scaffolds. Induced regions assigned to state 1 are much more likely to be on a major scaffold than induced regions assigned to state 2 (Odds ratio = 14.3, Fisher's Exact Test *P*-value = 4.85 × 10^-12^). As expected, regions on minor scaffolds, irrespective of class, are much more likely to fail to be associated with an annotated gene (Odds ratio = 144.6, Fisher's Exact Test *P*-value < 2.2 × 10^-16^). However, the difference between minor and major scaffolds does not seem to fully explain the difference between state 1 and state 2, as even when restricted to just the major scaffolds expressed regions assigned to state 1 are more likely to be associated with genes (Odds ratio = 4.4, Fisher's Exact Test *P*-value = 0.0027). It could be that highly induced genes are more likely to have homologs in *D. melanogaster*, increasing the probability that those genes would be annotated in *D. virilis*. However, among just the regions that are associated with genes, it is actually state 2 that is more likely to have homologs in *D. melanogaster *(based on fuzzy reciprocal BLAST calls downloaded from FlyBase under the "Genomes FTP" section; Odds ratio = 3.60, Fisher's Exact Test *P*-value = 0.0176). Because of the difficulties in annotating genes on minor scaffolds, we have limited our primary analysis to the 33 expressed regions in state 1, plus the 166 expressed regions in state 2 that are on major scaffolds: this sample of 199 expressed regions includes 101 that can be associated with an annotated gene, as described above, and 98 that cannot.

### Sample preparation and iTRAQ labeling for proteomic studies

We extracted hemolymph from approximately 200 *D. virilis *females 24 hours after infection with a mixed culture of *E. faecalis *and *S. marcescens *(infected sample) and 200 uninfected controls (uninfected sample). Hemolymph extracts were spun for 10 minutes at low speed to pellet cellular material. Equal amounts of each sample (40 μg protein) were then aliquoted in duplicate, reduced, cysteine-blocked and digested by trypsin. Each aliquot was labeled with a different iTRAQ tag according to the manufacturer's instructions (document #4351918A and 4350831C downloaded from , Applied Biosystems, Foster City, CA). The 114 and 115 tags were used to label the peptides in the two identical samples from control (uninfected) sample and the 116 and 117 tags were used for two extracts from the infected sample. After labeling, the four samples were pooled and subjected to cation exchange chromatography as described below.

### Strong Cation Exchange Fractionation

Strong cation exchange (SCX) fractionation was completed using an Agilent 1100 HPLC with UV detector (Agilent Technologies, Inc. Santa Clara, CA). The tryptic peptides labeled with iTRAQ tags were reconstituted in buffer A (10 mM potassium phosphate pH 3.0, 25% ACN), prior to SCX LC. The samples (~400 μg) were loaded onto a PolyLC Polysulfoethyl A column (2.1 mm × 150 mm) purchased from PolyLC Inc. (Columbia, MD). Buffer B was composed of 10 mM potassium phosphate pH 3.0, 25% ACN with 1 M KCl. Sample fractionation was completed using the gradient 0% B, 15 min, 0–25% B in 40 min, 25–50% B in 10 min and hold 50% B for 10 min. During this elution, forty fractions were collected at a flow rate of 200 μl/min on the basis of the UV trace at 214 nm. Several fractions were pooled post-collection to yield a total of 10 sample containing fractions. Salts were removed via solid phase extraction using Waters SepPak C18 cartridge (Waters, Milford, MA) and purified peptide fractions were dried and reconstituted in 2% ACN, 0.05% formic acid and injected on the nLC-MS/MS.

### Reverse-Phase Separation and Tandem Mass Spectrometry

The 10 SCX fractions were partially evaporated to remove ACN and desalted by solid phase extraction. All SPE-extracted and gel-extracted peptide samples were reconstituted in 50 μl of 0.1% formic acid with 2% acetonitrile prior to mass spectrometry (MS) analyses. NanoLC was carried out by an LC Packings Ultimate integrated capillary HPLC system equipped with a Switchos valve switching unit (Dionex, Sunnyvale, CA). An aliquot of peptide fractions (5.0 μl) were injected using a Famous auto sampler onto a PepMap C18 trap column (5 μm, 300 μm × 5 mm, Dionex) for on-line desalting and then separated on a PepMap C-18 RP nano column, eluted in a 90-minute gradient of 5% to 40% acetonitrile in 0.1% formic acid at 250 nl/min. The nanoLC was connected in-line to a hybrid triple quadrupole linear ion trap mass spectrometer, 4000 Q Trap from ABI/MDS Sciex (Framingham, MA) equipped with Micro Ion Spray Head ion source. MS data acquisition was performed using Analyst 1.4.2 software (Applied Biosystems) in the positive ion mode for information dependant acquisition (IDA) analysis. The nanospray voltage was 2.0 kV used for all experiments in positive ion mode. Nitrogen was used as the curtain (value of 10) and collision gas (set to high) with heated interface at 150°C. The declustering potential was set at 50 eV and Gas1 was 15 (arbitrary unit). In IDA analysis, after each survey scan for *m/z *400 to *m/z *1550 and an enhanced resolution scan, the three highest intensity ions with multiple charge states were selected for tandem MS (MS/MS) with rolling collision energy applied for detected ions based on different charge states and *m/z *values.

### Data analysis and protein identifications

MS/MS spectra generated from nanoLC/ESI-based IDA analyses were interrogated using ProteinPilot™ software 2.0 (Applied Biosystems, Foster City, CA) for database search against the FlyBase FB2008_06 *D. virilis *peptides database by Paragon method. The default setting for trypsin with MMTS modification of cysteine and a methionine oxidation was used for quantitative processing and rapid ID. The protein identifications are reported with total ProtScore >1.3 for each protein representing > 95% statistical significance in ProteinPilot. In order to estimate abundance ratios and statistical significance for each identified protein, we created custom R scripts (available upon request) that extended the ProteinPilot statistical approach to account for the technical replication included in our experiment. Briefly, for each peptide we calculate an average infected/uninfected ratio as well as an average error (based on the reported ratio error for each of the two technical replicates per treatment). These ratios are then averaged using 1/Error as a weighting factor, and significance is determined using the weighted standard deviation as described in the ProteinPilot manual (ABI).

For the estimated abundance analysis of each identified protein, the MS/MS data were also submitted to Mascot 2.2 for database searching using in-house licensed Mascot local server against the same FlyBase FB2008_06 *D. virilis *peptides database with one missed cleavage site by trypsin allowed and iTRAQ-4-plex quantitation. The peptide tolerance was set to 1.2 Da and MS/MS tolerance was set to 0.6 Da. MMTS modification of cysteine and iTRAQ modification of N-terminal and lysine were fixed and a methionine oxidation and iTRAQ modification of tyrosine were set as variable modifications. Only significant scores for the peptides defined by Mascot probability analysis  greater than "identity" were considered for the protein identifications. The exponentially modified protein abundance index (emPAI) is obtained for each identified protein from Mascot searching and the corresponding protein content in mol percent is calculated as mol % = emPAI/Σ(emPAI) * 100 as reported previously [40]. For our analysis, we focus on the 144 proteins identified by both MASCOT and ProteinPilot, for which we can calculate both a infected/uninfected ratio and emPAI.

## Authors' contributions

TBS conceived and design the experiment, analyzed the data, and wrote the manuscript. AGC conceived and designed the experiment, coordinated data collection, and edited the manuscript. All authors read and approved the final manuscript.

## Supplementary Material

Additional File 1 **Expressed regions identified in *D. melanogaster***. Coordinates and induction state of all 4,615 expressed regions identified in *D. melanogaster*. Click here for file

Additional File 2 **Expressed regions identified in *D. virilis***. Coordinates and induction state of all 6,737 expressed regions identified in *D. melanogaster*. Click here for file

Additional File 3 **Induced regions associated with genes in *D. melanogaster***. Induction states and functional annotations of the 490 induced genes in *D. melanogaster*. Click here for file

Additional File 4 **Induced regions associated with genes in *D. virilis***. Induction states and functional annotations of the 95 induced genes in *D. virilis*. Click here for file

Additional File 5 **Proteins in the *D. virilis *hemolymph**. List of proteins that significantly change concentration after infection in the *D. virilis *hemolymph (nominal P < 0.01 significance cutoff). Click here for file
